# Yogic breathing when compared to attention control reduces the levels of pro-inflammatory biomarkers in saliva: a pilot randomized controlled trial

**DOI:** 10.1186/s12906-016-1286-7

**Published:** 2016-08-18

**Authors:** Waleed O. Twal, Amy E. Wahlquist, Sundaravadivel Balasubramanian

**Affiliations:** 1 Department of Regenerative Medicine and Cell Biology, Medical University of South Carolina, Charleston, SC 29425 USA; 2 Department of Public Health Sciences, Medical University of South Carolina, Charleston, SC 29425 USA; 3 Department of Radiation Oncology, Medical University of South Carolina, Charleston, SC 29425 USA

**Keywords:** Yoga, Yogic breathing, Thirumanthiram, Pranayama, Salivary biomarkers, Cytokines, Multiplex, Pro-inflammatory, Stress, Tamil

## Abstract

**Background:**

Self-report measures indicate that Yoga practices are perceived to reduce stress; however, molecular mechanisms through which YB affects stress are just beginning to be understood. While invasive sampling such as blood has been widely used to measure biological indicators such as pro-inflammatory biomarkers, the use of saliva to measure changes in various biomolecules has been increasingly recognized. As Yoga practice stimulates salivary secretion, and saliva is considered a source of biomarkers, changes in salivary cytokines before and after Yogic breathing exercise as specified in an ancient Tamil script, Thirumanthiram, were examined using a Cytokine Multiplex to compare to Attention Control (AC) group.

**Methods:**

Twenty healthy volunteers were randomized into two groups stratified by gender (*N* = 10 per YB and AC groups); The YB group performed two YB exercises, each for ten minutes, for a total of twenty minutes in a single session as directed by a trained Yoga instructor. The AC group read a text of their choice for 20 min. Saliva was collected immediately after YB training at 0, 5, 10, 15 and 20 min and analyzed by Multiplex enzyme linked immunosorbent assay (ELISA).

**Results:**

The levels of interleukin (IL)-1β, IL-8, and monocyte chemotactic protein −1 (MCP-1) were significantly reduced in YB group when compared to AC group. The level of reduction of IL-8 was significant at all time points tested, whereas IL-1β showed reduction at 15 and 20 min time points (*p* < 0.05), and MCP-1 level was marginally different at 5–20 min. There were no significant differences between YB and AC groups in the salivary levels of IL-1RA, IL-6, IL-10, IL-17, IP-10, MIP-1b, and TNF-α.

**Conclusions:**

These data are the first to demonstrate the feasibility of detecting salivary cytokines using multiplex assay in response to a Yoga practice. This study was registered in Clinical Trials.gov # NCT02108769.

**Electronic supplementary material:**

The online version of this article (doi:10.1186/s12906-016-1286-7) contains supplementary material, which is available to authorized users.

## Background

Pro-inflammatory cytokines and chemokines are implicated in several diseases and therefore are potential targets for molecular therapy. While non-pharmacological methods of stress reduction have long been in practice among several cultures, recently such practices are gaining acceptance worldwide. Translating information on stress reduction from ancient cultures into modern scientific literature is important to preserve and propagate such wellness practices [1]. Thirumanthiram is an ancient Tamil text written by Saint Thirumoolar who was contemporary to Saint Patanjali [2, 3]. Thirumanthiram (*Thiru* means holy or sacred or noble, *manthiram* means chant or mantra or hymn or *sutra*) consists of over 3000 poems referring to various practices including Ethics, Devotion, and Yoga. In this anthology there are 14 poems (Numbers 564 through 577) dedicated to the practice of Yogic Breathing (YB, *Pranayamam* or *Pranayama*). These 14 poems speak in depth about the benefits of YB and explain how breathing is an important connection between mind and body.

YB is one of the several practices within the broad field of Yoga and is known to cause key changes within mind and body including blood pressure reduction, heart rate variability changes, and breathing frequency reductions [4], predominant abdominal/diaphragmatic breathing [5–7], improved cognitive functions (e.g., mental alertness and reduced cognitive failure) [8, 9], increased bimanual dexterity and visuo-motor co-ordination [10], stress and symptom reduction in diseases such as cancer [11]. Behavioral practices such as relaxation and exercise could bring about meaningful molecular changes among practitioners [12, 13]. Although Yoga practitioners from various schools widely practice YB, the techniques specified by Thirumoolar have not yet been studied for their physiological effects or changes in biomarkers. This study focused on the YB exercise specified in Poem 568 of Thirumanthiram (named Thirumoolar Pranayamam, TMP) that includes an inhalation (*Purakam*), breath-holding (*Kumbakam*), and exhalation (*Resakam*) for specified time periods [3]. To measure the changes in physiological responses to TMP, saliva was used to measure biomarkers. Saliva is an easily accessed non-invasive biologic sample rich in various biomarkers including proteins, peptides, metabolites, mRNA, DNA, lipids, and miRNA [14–18]. We recently reported that the practice of TMP increases salivary nerve growth factor [19], and caused wide range alteration to salivary proteome [20].

Based on the ability of YB to reduce stress and because salivary biomarkers could be utilized to assess stress, it was hypothesized that the practice of TMP would reduce the salivary expression of pro-inflammatory biomarkers. The results indicate that YB practice as specified in Thirumanthiram in fact reduced some of the key biomarkers implicated in stress and inflammation. Identification of molecular changes in easily accessible saliva could increase the ability of correlating subjective responses with biochemical measures in clinical trials involving Yoga and other mind-body practices.

## Methods

### Human subjects

This study used the salivary samples collected from a recent clinical trial [19, 20] that involved a total of twenty healthy volunteers (male and female), aged 18 and above. The exclusion criteria were: breathing problems (inability to breath through nostrils, chronic bronchitis, emphysema and asthma), speech problems that would prevent chanting, inability to listen and follow study exercise, sinus congestion, Sjogren’s syndrome, chronic dry mouth due to medication or other conditions, and use of anti-cholinergic medications. Informed consent was obtained from each subject after initial interview. This trial was approved by the institutional ethics committee, Health Sciences South Carolina Institutional Review Board (Approval number: PRO#24336), and registered in Clinical Trials.gov (# NCT02108769).

**Fig. 1:**
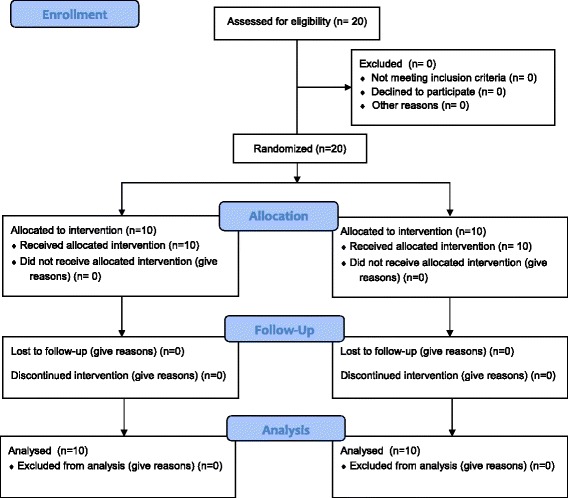
CONSORT flow chart

Enrolled participants were randomized to one of two conditions: Yogic Breathing (YB) arm versus the Attention Control (AC) arm (Fig. 1. CONSORT Flow Chart). Randomization was stratified by gender to ensure equal gender distribution in the two experimental groups (YB vs. AC). All participants were tested for YB or AC intervention one-on-one with a trained Yoga instructor. Just prior to intervention and sample collection, the Yoga instructor taught each participant in the YB group how to perform YB, which consisted of a combination of 10 min of Om chanting (*Pranava Pranayama*) followed by 10 min of TMP as described previously [19, 20]. Briefly, the participants were taught to inhale through one nostril for two counts, hold the breath for eight counts, and exhale for four counts. This cycle was repeated for 10 min. The AC group performed quiet reading for the same period in independent one-on-one sessions. The Attention Control group was used as a control group for Yogic Breathing as in previous studies on mind-body practices [21]. Salivary samples were collected immediately following the training at the beginning of the protocol (Time 0) and at 5, 10, 15 and 20 min from both groups of participants. Saliva was naturally allowed to accumulate in the oral cavity, and the participant discharged (1–4 mL) into the 15 mL polystyrene specimen tube with lid. Samples were immediately cooled on ice and stored at −80°C within 15 min of collection until analysis.

### Cytokine multiplex assay

Ten stress related cytokines (IL-1β, IL-1RA, IL-6, IL-8, IL-10, IL-17, IP-10, MCP-1, MIP-1b, and TNF-alpha) were analyzed by using a commercial kit (Bio-Plex Pro Human Cytokine Group I 10-plex Assay, Catalog Number Y5002UN3DT, Bio-Rad) as per the manufacturer’s instructions. Fifty microliters of cleared saliva from each time point from all AC and YB participants were subjected to multiplex assay in duplicate. Cytokine levels were expressed as pg/mL based on the average fluorescent intensities as measured using Bio-Plex 200 Multiplex System (Bio-Rad) at the MUSC Proteogenomics facility.

### Statistical analysis

Analyses were conducted using SAS software, Version 9.3 of the SAS System for Windows (Copyright © 2002–2010 SAS Institute Inc., Cary, NC, USA). Linear mixed models with an Auto Regressive order 1 correlation structure were used to study the change in cytokine levels over time (0, 5, 10, 15, and 20 min) between YB and AC groups. Group and time main effects were included in the models. Due to the small sample size and a lack of ability to detect significant interactions between group and time, these were not included in this pilot analysis. For cytokine values that were out of range (OOR), the minimum (or maximum) standard values were imputed, as appropriate. If values could be extrapolated beyond the standard values, then these values were used instead of the minimum/maximum standard values. Heat-maps were used to visualize any patterns of cytokine levels between the two groups. P-values less than 0.05 were considered statistically significant.

### Availability of supporting data

Data obtained from all study participants at individual time points for all ten different cytokines measured in the current study are presented as Additional file 1: Table S1.

## Results

A total of 20 participants were enrolled in the study: 10 males and 10 females. There were 50% males and 50% females, and within each gender 50% each were randomized to AC and YB groups. The median age and interquartile range (IQR) for each group are presented in Table 1. There were no dropouts or adverse reactions reported due to the study exercises.

**Table 1:** Median age and interquartile range (IQR) for each group of participants

	AC	YB
Male, n (%)	5 (50%)	5 (50%)
Age, median (IQR)	33 (13)	29 (9)
Female, n (%)	5 (50%)	5 (50%)
Age, median (IQR)	27 (11)	30 (5)

Salivary cytokine levels were analyzed over time (time points 0, 5, 10, 15 and 20 min) in YB and AC groups. The analysis indicated that the cytokines IL-1β, IL-8 and MCP-1 showed significant differences between groups and changed significantly over time. Overall, IL-1β, IL-8, and MCP-1 levels were significantly lower for YB than for AC participants. In general, the YB group also had more stable values over time than the AC group. All other cytokines analyzed (IL-1RA, IL-6, IL-10, IL-17, IP-10, MIP-1b, and TNF-alpha) did not show any significant differences between groups at all time points (Additional file 1: Table S1).

**Fig. 2:**
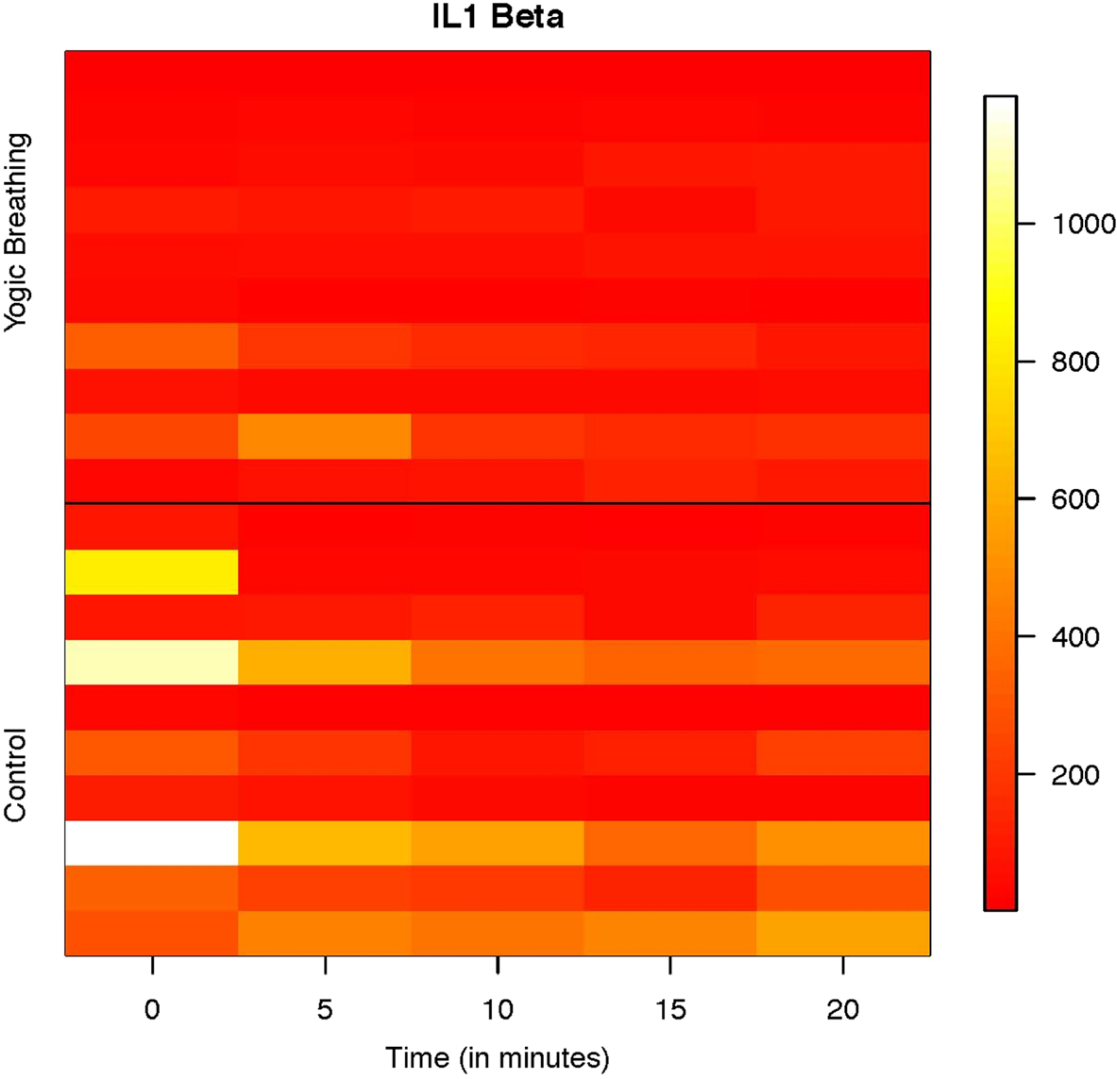
Change in salivary IL-1β level in Attention Control and Yogic breathing groups. Salivary samples from Attention Control (Control) and Yogic Breathing group were analyzed in Multiplex ELISA (Bio-Plex Pro Human Cytokine Group I 10-plex Assay, Bio-Rad). Individual data points on IL-1β (pg/mL) from all subjects were used to produce the heat-map

Figure 2 shows the heat-map at individual time points for IL-1β from all individuals in the AC and YB groups. At each time point the level of IL-1β was significantly reduced in YB group when compared with AC group (Table 2). It is interesting to note that the values of IL-1β were diminished in YB group at the 0 min mark, which is believed to be because the YB exercises were taught to the subjects just prior to taking the measurements. This suggests that mere practicing/learning for a brief period (typically around 5 min) could reduce the levels of IL-1β. It can also be observed from at least some of the individuals from AC group that there is a trend to reduce the IL-1β levels by mere quiet reading. However, these levels were significantly higher than those observed with YB group at each time point. These data indicate that IL-1β level could be significantly reduced by the YB exercise.

**Table 2:** Changes in salivary IL-1β, IL-8 and MCP-1 levels in Yogic Breathing (YB) group when compared with Attention Control (AC) group

	0 min		5 min		10 min		15 min		20 min	
	AC	YB	AC	YB	AC	YB	AC	YB	AC	YB
IL-1β (pg/mL)	432.40 (440.99)	87.04 (110.64)	231.69 (248.58)	95.43 (142.79)	184.96 (200.35)	62.96 (60.07)	149.98 (172.63)	66.12 (52.34)	215.10 (211.72)	66.18 (49.24)
IL-8 (pg/mL)	1812.13 (2304.86)	364.68 (375.22)	539.79 (336.52)	150.72 (101.32)	476.28 (275.59)	159.05 (142.59)	426.33 (283.75)	145.47 (100.20)	845.10 (915.08)	196.05 (149.71)
MCP1 (pg/mL)	329.38 (232.63)	143.03 (167.12)	201.90 (95.70)	91.21 (94.37)	186.81 (111.70)	103.95 (113.81)	149.20 (67.97)	97.47 (110.70)	275.95 (211.78)	162.32 (199.17)

Saliva samples from both groups were collected at 0, 5, 10, 15 and 20 min were analyzed multiplex assay as explained under Methods. Pooled data (with standard deviation in parentheses) expressing levels of IL-1β, IL-8 and MCP-1 levels (pg/mL) from all the individuals from YB and AC groups at each time point were used for statistical analysis using linear mixed models with Auto Regressive order 1 correlation structure

**Fig. 3:**
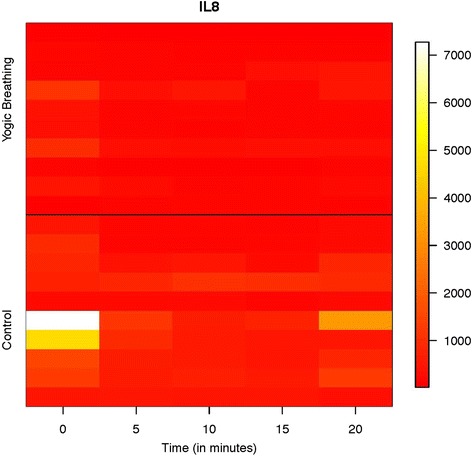
Change in salivary IL8 level in Attention Control and Yogic breathing groups. Change in IL-8 level in Control and Yogic breathing groups. Salivary samples from Attention Control (Control) and Yogic Breathing group were analyzed in Multiplex ELISA (Bio-Plex Pro Human Cytokine Group I 10-plex Assay, Bio-Rad). Individual data points on IL8 (pg/mL) from all subjects were used to produce the heat-map

Changes in the level of IL-8 are shown in a heat-map in Fig. 3 and in Table 2. It is clear from these data that YB could reduce the level of IL-8 at all time points. While AC could also show a trend of reduction, the magnitude of IL-8 reduction in YB group was 3–6 orders lower than the AC group. This indicates that salivary expression of IL-8 could be significantly reduced by YB practice.

**Fig. 4:**
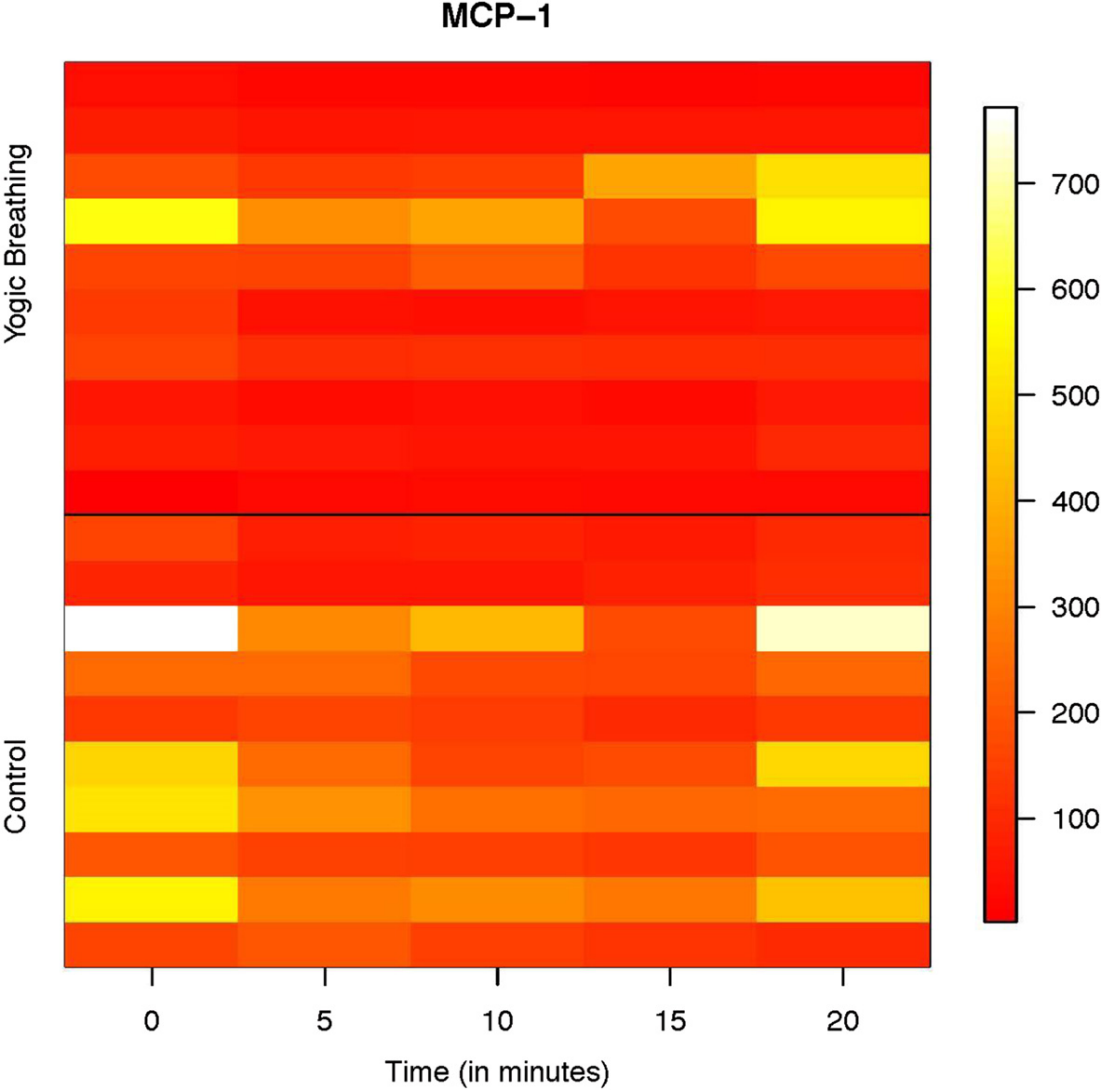
Change in salivary MCP-1 level in Attention Control and Yogic breathing groups. Change in MCP-1 level in Control and Yogic breathing groups. Salivary samples from Attention Control (Control) and Yogic Breathing group were analyzed in Multiplex ELISA (Bio-Plex Pro Human Cytokine Group I 10-plex Assay, Bio-Rad). Individual data points on MCP-1 (pg/mL) from all subjects were used to produce the heat-map

Apart from these two cytokines, there were marginally significant alterations in the chemokine monocyte chemotactic protein (MCP-1) also known as chemokine (C-C motif) ligand 2 (CCL2). While the AC group did not show any decline in salivary expression of MCP-1 as in the case of other cytokines (IL-1β and IL-8), the YB group showed a significant reduction in MCP-1 level in saliva (Fig. 4 and Table 2). The overall results for the main effects of each model are provided in Table 3, and the interaction plot showing changes in all the three cytokines with time in both YB and AC groups is shown in Additional file 2: Figure S1. The heat maps provide more information than the interaction plots for this study because they showed individual level and group level values for each of the cytokines while an interaction plot with means/SDs summarized the data per group and did not provide the individual level data. These results, in combination with the pooled data seen in Table 2 and the heat-maps for each of the significant cytokines, give a full picture on how these cytokines are changing between groups and over time.

**Table 3:** Results from the linear mixed model analyses for each cytokine for group (AC vs. YB) and time (0, 5, 10, 15, 20 min)

		F	*P*-value
IL-1β	Group^a^	6.22	0.02
	Time^b^	3.39	0.01
IL-1RA	Group^a^	0.08	0.8
	Time^b^	0.73	0.6
IL-6	Group^a^	0.41	0.5
	Time^b^	0.95	0.4
IL-8	Group^a^	7.96	0.01
	Time^b^	4.05	0.005
IL-10	Group^a^	0.17	0.7
	Time^b^	1.32	0.3
IL-17	Group^a^	1.27	0.3
	Time^b^	1.52	0.2
IP-10	Group^a^	0.35	0.6
	Time^b^	3.54	0.01
MCP-1	Group^a^	4.43	0.0495
	Time^b^	6.19	0.0002
MIP-1b	Group^a^	0.02	0.9
	Time^b^	1.68	0.2
TNF-alpha	Group^a^	0.00	>0.9
	Time^b^	1.34	0.3

The overall F statistic for each main effect is provided as well as the significance level (*p*-value); the degrees of freedom for the individual main effects were constant across all models

^a^Numerator df = 1, denominator df = 18

^b^Numerator df = 4, denominator df = 76

## Discussion

This is the first time a multiplex assay format has been used for the determination of salivary cytokines from a Yoga based exercise. These results show a significant reduction in the levels of IL-1β, IL-8 and MCP-1 in the YB group when compared to the AC group. IL-1β is implicated in stress and inflammation in various diseases. Cleavage by caspases-1 and 8 activates IL-1β for subsequent induction of cyclooxygenase-2 in the central nervous system, which is involved in stress and pain perception [22]. Also, at the systemic level, the increased IL-1β level potentiates a variety of pro-inflammatory cellular processes such as proliferation, migration, apoptosis and differentiation. Although these processes are important for host defense mechanisms, higher levels of IL-1β is deleterious as shown in several diseases including cancer (reviewed in [23]). Therefore IL-1β is pursued as a clinical target (for example, Canakinumab [24], which is used for treating autoinflammatory syndromes and in clinical trial for chronic obstructive pulmonary disease).

IL-8 is a key mediator of inflammatory processes during various stress conditions involving oxidative stress. Elevated levels of IL-8 is associated with poor prognosis of breast cancer [25] and in psychosomatic disorders [26, 27]. Although targeting IL-8 pharmacologically is perceived to be important, there is no targeted therapy specifically directed against IL-8. Under these circumstances the current study shows that Yogic Breathing could be an effective non-pharmacological approach to regulate the level of this cytokine.

During tissue injury or infection, MCP-1 recruits key immune cells including monocytes, memory T cells, and dendritic cells; however, the level of MCP-1 is elevated in chronic pathological conditions. The level of salivary MCP-1 is used as a biomarker of various physiological dysfunctions including coronary heart disease and post-traumatic stress disorder (PTSD) where MCP-1 is secreted in large quantities by monocytes recruited in response to chronic endothelial inflammation [28]. These studies also indicate that there was a correlation between the salivary levels of MCP-1 and PTSD symptoms, depression, and anxiety. Interestingly, all the three biomarkers decreased by YB in the present study were reported to be significantly higher in asthmatic individuals when compared to controls [29]. It is important to note that Yoga practices including YB exercises are increasingly being used in the control of asthma [30, 31]. This is the first study ever to find a relationship between Yoga practice and MCP-1. Several studies found key association between elevated levels of MCP-1 and increased severity of pathogenesis including Alzheimer’s disease (AD) [32]. While MCP-1 is increased in AD patients, these observations showing a decrease in MCP-1 could be important for the management of AD. In addition to a reduction in MCP-1, an elevation in nerve growth factor [19] upon YB practice could be at least two key molecular targets elicited in response to YB towards the management of AD.

Identification of these salivary biomarkers in response to a mind-body practice may serve as an important point towards establishing molecular mechanisms associated with these practices. Recently it was shown that relaxation response could produce transcriptomic level changes in several molecules associated with inflammation, immune response, and energy metabolism [12], which correlates with previous studies showing proteomic level changes following YB [20], and changes in pro-inflammatory biomarkers in the present study. Although the mechanism by which breath regulation could reduce salivary pro-inflammatory biomarkers is not clear, it is speculated that the YB exercises administered in the present study might have elicited a mild hypercapnia similar to earlier observations following slow breathing [7], certain slow Yogic breathing exercises [33–35], and breath holding [36]. Hypercapnia has been used as a therapeutic strategy in clinical settings such as lung injury [37] and hypothesized to be useful in cardiac arrest [38]. It is suggested that hypercapnia reduces inflammation through modulating NFkB mediated inflammatory signaling [39, 40]. This observation needs to be correlated with the studies of Bhasin et al. [12] where the transcriptome that showed significant reductions during relaxation response was associated with NFκB signaling node, and the present data showing a reduction in all three biomarker expressions is reported to be mediated through NFκB signaling in numerous cellular contexts [41, 42]. Therefore, it is hypothesized that hypercapnia could be a possible mediator of reducing inflammatory biomarker release following YB practice. Further studies are needed to understand this relationship. The immunological effects of observing the reduced levels of cytokines could be as a result of sequestration of cytokines in the salivary milieu. Our earlier published work shows that several proteins with broad agglutinating/sequestering functions are induced upon Yogic breathing exercise. These proteins include clusterin, CRISP3, DMBT1, and mucin 7. It has been proposed that such cytokine or growth factor sequestering function is important in cancer prevention and control [43]. This could be one of the ways by which a short term Yogic breathing training reduces cytokine levels in saliva, however requires further experimental support.

This study is innovative in the following respects: 1) Use of salivary pro-inflammatory biomarkers using multiplex assay format for the analysis of Yoga outcomes, and 2) This is the first time that Thirumoolar Pranayamam from an ancient Tamil literature was studied for its effects on the salivary expression of pro-inflammatory biomarkers. While this is the first study approaching Thirumoolar Pranayamam for its anti-inflammatory biomarkers, earlier studies have shown evidence for the stress reducing ability of Pranayama methods. The other Pranayama method used in the present study, Om chanting also called *Pranava Pranayama*, is an ancient cultural practice involving breath regulation thought to promote overall physical and emotional well-being [8, 44]. Previous studies show that this type of breath regulation could exert the following effects: caused significant reduction in heart rate and blood pressure in hypertensive patients within 5 min of practice [45], which incidentally correlates with the stress biomarker changes seen in 5 min of practice in the present study; and, increased cutaneous peripheral vascular resistance, which is a sign of increased mental alertness even while being physiologically relaxed [44]. Taken together, this data provides a biochemical support for the observed relaxation experience with the practices of regulated breathing.

Salivary cytokines have recently been tested in various stress related conditions including diabetes [46], and as a measure of immune function [47]. Yoga studies so far have relied mainly on subjective measures (e.g., self report of quality of life and stress) to determine the effectiveness of Yoga intervention [48]. Recently, gene expression studies in peripheral blood following 8 weeks of relaxation techniques (including meditation, Yoga, and breath regulation) identified a number of genes involved in metabolism, energy regulation, and inflammation [12]. We reported that salivary nerve growth factor could be induced [19], and salivary proteome could be altered [20] by these YB exercises. This further supports the hypothesis that YB could acutely alter the levels of salivary biomolecules with key biological functions immediately after practice, and this might be important not only in the oral cavity but might also have systemic effects. Some limitations of the present study include the small sample size, and the cytokine levels at Time 0 being taken after learning the breathing exercise in the YB group as opposed to prior to learning the technique. Because participants had already learned the technique, the cytokine levels may not represent a true baseline measurement.

## Conclusion

For the first time, data showing that a single 20 min session of Yogic Breathing practice could reduce the levels of key pro-inflammatory biomarkers in saliva. Future studies using saliva as a source of biomarkers in individuals during the practice of various Yoga techniques could help deduce molecular mechanisms through which these Yoga techniques may work.

## Additional files

Additional file 1: Table S1. Changes in salivary levels of pro-inflammatory biomarker panel in Yogic Breathing (YB) group when compared with Attention Control (AC) group. Saliva samples from both groups were collected at 0, 5, 10, 15 and 20 min were analyzed multiplex assay as explained under Methods. Data expressing levels of IL-1β, IL-1RA, IL-6, IL-8, IL-10, IL-17, IP-10, MCP-1, MIP-1b, and TNF-alpha (pg/mL) from all the individuals from YB and AC groups at each time point are presented. (XLSX 66 kb) Additional file 2: Figure S1. Interaction plots showing means/SDs for salivary IL-1β, IL-8 and MCP-1 levels (pg/mL) over time (minutes) in Attention Control (red line) and Yogic breathing (black line) groups. (DOCX 62 kb)

## Abbreviations

AC: Attention control
AD: Alzheimer’s disease
CCL2: Chemokine (C-C-motif) ligand 2
DMBT1: Deleted in malignant brain tumors-1
ELISA: Enzyme linked immunosorbent assay
IL1-RA: Interleukin-1 receptor antagonist
IL: Interleukin
IP10: Interferon gamma-induced protein 10
IQR: Interquartile range
MCP1: Monocyte chemotactic protein-1
MIP-1b: Macrophage inflammatory protein 1-beta
NGF: Nerve growth factor
OOR: Out of range
PTSD: Post-traumatic stress disorder
TMP: Thirumoolar pranayamam
TNFα: Tumor necrosis factor alpha
YB: Yogic breathing
